# Influence of the Constitutive Model for Shotcrete on the Predicted Structural Behavior of the Shotcrete Shell of a Deep Tunnel

**DOI:** 10.3390/ma10060577

**Published:** 2017-05-25

**Authors:** Matthias Neuner, Magdalena Schreter, David Unteregger, Günter Hofstetter

**Affiliations:** Unit for Strength of Materials and Structural Analysis, Institute of Basic Sciences in Engineering Science, Innsbruck University, Technikerstr. 13, Innsbruck A-6020, Austria; Magdalena.Schreter@uibk.ac.at (M.S.); david.unteregger@uibk.ac.at (D.U.); Guenter.Hofstetter@uibk.ac.at (G.H.)

**Keywords:** shotcrete, tunneling, finite element model, numerical simulation, constitutive model, damage model, plasticity model

## Abstract

The aim of the present paper is to investigate the influence of the constitutive model for shotcrete on the predicted displacements and stresses in shotcrete shells of deep tunnels. Previously proposed shotcrete models as well as a new extended damage plasticity model for shotcrete are evaluated in the context of 2D finite element simulations of the excavation of a stretch of a deep tunnel by means of the New Austrian Tunneling Method. Thereby, the behavior of the surrounding rock mass is described by the commonly used Hoek–Brown model. Differences in predicted evolutions of displacements and stresses in the shotcrete shell, resulting from the different shotcrete models, are discussed and simulation results are compared to available in situ measurement data.

## 1. Introduction

The use of shotcrete plays an essential role within the New Austrian Tunneling Method (NATM). Subsequent to each excavation step, shotcrete is directly sprayed onto the surrounding rock mass, forming a supporting structure which is usually loaded already several hours after casting due to further advance of the tunnel. The prediction of the structural behavior of the rock-support system is a complex time-dependent problem and requires realistic constitutive modeling of both shotcrete and the surrounding rock mass. However, in numerical simulations, the constitutive behavior of shotcrete is often approximated using simple material models, like linear-elastic models. In contrast to normal concrete, only a few constitutive models for shotcrete representing the highly nonlinear, time-dependent material behavior have been proposed in the literature so far. Frequently used representatives of the latter are:

- The viscoplastic shotcrete model by Meschke [1]: Nonlinear mechanical behavior of shotcrete is described on the basis of the multisurface viscoplasticity theory using an associated flow rule. A hardening Drucker–Prager model is used for predominant compressive stress states and mixed stress states and a softening Rankine criterion for predominant tensile stress states to model cracking. The evolution of stiffness is described by hyperelastic constitutive relations. Aging of shotcrete is considered by evolution laws for the Young’s modulus, the uniaxial compressive strength and the uniaxial tensile strength. Shrinkage of shotcrete is taken into account on the basis of the semi-empirical model proposed by Bažant and Panula [2]. Creep of shotcrete is modeled by a Duvaut–Lions type viscoplastic formulation.
- The viscoelastic-plastic shotcrete model by Schädlich and Schweiger [3]: Nonlinear mechanical behavior of shotcrete is described on the basis of multisurface plasticity theory using a non-associated flow rule. A hardening and softening Mohr–Coulomb model is used for predominant compressive stress states and mixed stress states and a softening Rankine criterion for predominant tensile stress states to model cracking. Aging of shotcrete is considered by evolution equations for stiffness and strength. Shrinkage of shotcrete is taken into account on the basis of the model proposed by the American Concrete Institute (ACI) committee 209 [4]. Nonlinear creep of shotcrete is modeled on the basis of the theory of viscoelasticity.
- The thermo-chemo-mechanical shotcrete model proposed by Hellmich et al. [5]: An alternative to single-field shotcrete models are multi-field models. Considering the dependency of shotcrete properties on the degree of hydration, i.e., on the time- and temperature-dependent chemical reaction between cement and water, it is based on thermo-chemo-plasticity theory along the lines of the thermo-chemo-mechanical framework, developed by Ulm and Coussy [6]. This model was developed further in [7] to consider early age cracking of shotcrete.
- The shotcrete damage plasticity (SCDP) model [8]: It is based on (i) the damage plasticity model by Grassl and Jirásek [9] to describe hardening and softening material behavior; (ii) the solidification theory by Bažant and Prasannan [10] to represent aging and creep; and (iii) the shrinkage model by Bažant and Panula [2]. To account for the early age behavior of shotcrete, a modification of the solidification theory is employed. Time-dependent material properties are represented by evolution laws for material strength and for ductility.

Applications of shotcrete models in numerical simulations of tunneling can be found, e.g., in the following papers:

- Pöttler [11] used a linear-elastic shotcrete model in the simulation of the advance of a shallow railway tunnel. In order to take into account the influence of time-dependent shotcrete behavior due to hydration and creep and the time-dependent process of tunneling in a simplified manner, a reduced Young’s modulus of shotcrete, denoted as Hypothetical Modulus of Elasticity (HME), was employed. The HME was derived from a parameter study on a 2D finite element simulation of tunnel advance using a viscoelastic material model for shotcrete and it was concluded that a good approximation of the complex nonlinear time-dependent numerical model is achieved by a linear-elastic material model for shotcrete with an HME of 7000 MPa. However, the derivation of the HME was restricted to axisymmetric solutions and linear-elastic behavior of the rock mass.
- Meschke et al. [12] applied their viscoplastic shotcrete model in combination with an extended version of the soil model by DiMaggio and Sandler [13] in 3D finite element simulations of a shallow tunnel and compared the results to in situ measurement data. The tunnel was driven according to a partial excavation scheme, consisting of sequential excavation and securing of crown, bench and invert.
- Schädlich and Schweiger [14] applied their viscoelastic-plastic shotcrete model in 2D finite element simulations of the advance of a shallow tunnel with temporary side drift walls using a Mohr–Coulomb yield criterion for the surrounding soil. Focus was on the realistic prediction of the relaxation of bending moments in the shotcrete shell due to creep.
- Lackner and Mang [7] analyzed the stress state in the shotcrete shell during tunnel advance using an extended version of the thermo-chemo-mechanical shotcrete model by Hellmich et al. [5]. A hybrid method based on prescribed displacements at the rock-shotcrete interface, available from in situ measurement data, in combination with a structural model of the shotcrete shell, was used. Special attention was paid to cracking of shotcrete due to bending moments resulting from heterogeneous soil and rock conditions and due to tensile loading induced by shrinkage and thermal gradients. In contrast to isotropic softening models, two Rankine criteria in longitudinal and circumferential direction were used. Hence, the tensile strength was affected only by the respective crack direction.

Most of the case studies in the literature refer to tunnels with low overburden [7,11,12,14,15,16,17]. To the authors’ best knowledge, no comparison of constitutive models for shotcrete based on numerical simulations of tunneling has been presented so far. This is the motivation for complementing the comparative investigation of shotcrete models at material point level in [8] by a comparative investigation of the influence of the respective shotcrete models on the structural behavior of the shotcrete shell by means of a benchmark example of deep tunnel advance. In particular, the performance of selected shotcrete models, i.e., (i) the SCDP model [8]; (ii) the model by Schädlich and Schweiger [3]; (iii) the model by Meschke [1] and (iv) the approach proposed by Pöttler [11] are evaluated by means of 2D finite element simulations of deep tunnel advance, employing the commonly used Hoek–Brown model for describing the behavior of the surrounding rock mass.

The paper is organized as follows: in Section 2, the 2D initial boundary value problem, serving as the benchmark example for the simulation of deep tunnel advance, is presented and the material parameters of the constitutive models for shotcrete and rock mass are specified. In Section 3, simulation results on the basis of the respective shotcrete models, in combination with the Hoek–Brown model for the rock mass, are presented. The shotcrete models are compared by means of the predicted temporal evolution of displacements and stresses in the shotcrete shell. Finally, in Section 4, a summary and conclusions are presented.

## 2. Finite Element Model of Deep Tunnel Advance

A deep tunnel with circular cross-section, driven in Innsbruck quartz phyllite by the New Austrian Tunneling Method (NATM), is considered. The numerical model is derived from a stretch of the Brenner Basetunnel for which in situ measurement data is available. For a detailed description the reader is referred to [18]. To account for 3D effects due to the excavation process in 2D simulations, an equivalent plane strain model using the convergence confinement method as discussed in, e.g., [19,20], is developed.

### 2.1. Geometry and Boundary Conditions

**Figure 1:**
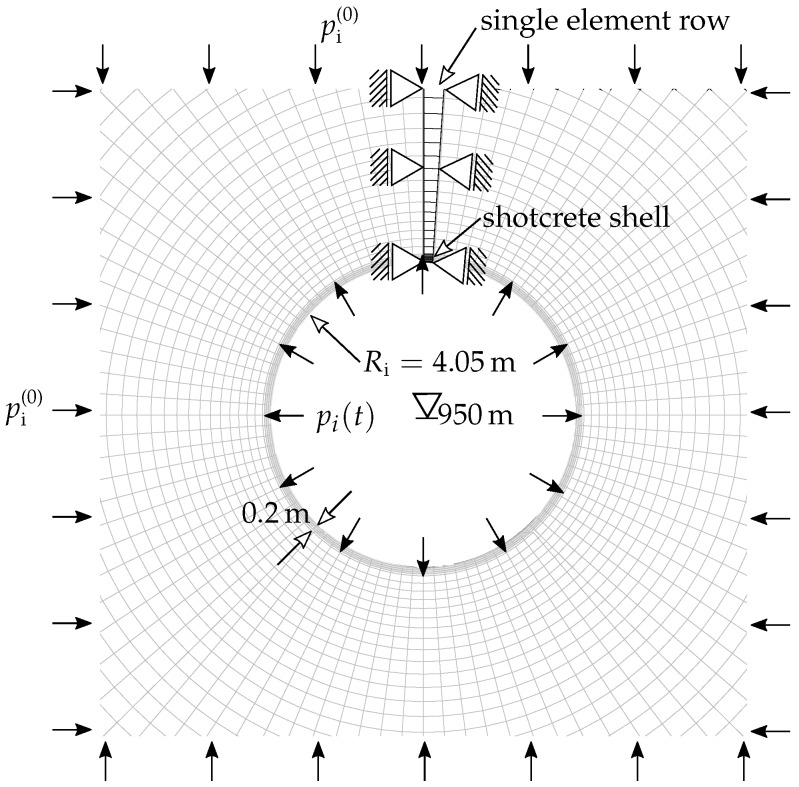
Schematic view of the benchmark problem together with the 2D axisymmetric finite element model.

The analyzed tunnel section is characterized by a circular excavation profile with a diameter of 8.5 m and an overburden of 950 m measured from the tunnel axis. The shotcrete shell has a thickness of 0.2 m. The prevailing initial geostatic stress state is assumed as a constant hydrostatic pressure of $p_{i}^{\left(0\right)}=25.7\mathrm{MPa}$ within the discretized domain of rock mass. It follows from the geometric properties, the assumed initial stress state, the boundary conditions and the full face excavation that axial symmetry with respect to the tunnel axis can be exploited leading to a reduced finite element model consisting of a single row of finite elements, highlighted in black in Figure 1, in contrast to the full 2D model, illustrated in Figure 1 in gray. The discretized domain covers 100 m of the surrounding rock mass around the tunnel axis. The rock mass and the shotcrete shell are discretized by 8-node quadrilateral continuum elements, the latter by four elements through the thickness. Along the tunnel perimeter, a pressure boundary condition is applied, which will be described in the next subsection. Homogeneous Dirichlet boundary conditions are prescribed perpendicular to the remaining part of the boundary of the single row of finite elements.

This simple numerical model, representing an ideal situation, has been chosen because it allows a straightforward comparison of the different shotcrete models regarding the time-dependent response of the shotcrete shell. Since the assumed axisymmetric conditions idealize the actual situation at the tunnel construction site, in addition to axial forces, bending moments in the shotcrete shells are present, which are reduced by cracking and creep (or relaxation) of the shotcrete. However, the influence of bending moments on the response of a shotcrete shell has been the focus of several previous studies, e.g., [7,14].

### 2.2. Simulation of Tunnel Advance

The influence of the advance of the tunnel face on the considered cross section in the 2D analysis is expressed by reducing the fictitious internal pressure $p_{i}(t)$ acting on the tunnel perimeter, illustrated in Figure 1, by means of

(1) $$p_{i}^{\left(0\right)}(t)=\left(1-\lambda \left(t\right)\right)\;\;p_{i}^{\left(0\right)}.$$

**Figure 2:**
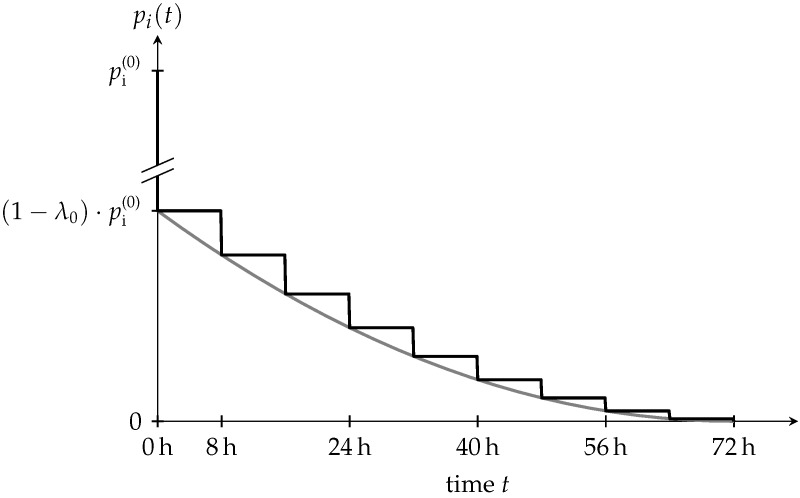
Stepwise stress release function $p_{i}(t)$ assumed in the finite element simulations (black curve), which is derived from the idealized parabolic stress release function, proposed by Pöttler [11] (gray curve).

Herein, *t* represents the time with t=0 referring to the installation of the shotcrete shell, $p_{i}^{\left(0\right)}$ is the initial hydrostatic pressure, which was specified in the previous subsection, and λ(t) denotes the stress release ratio ranging from 0% to 100%. The stress release ratio at installation of the shotcrete shell is defined by the initial stress release ratio $\lambda_{0}$. For the idealization of the 3D problem of tunnel advance by means of a 2D plane strain model, $\lambda_{0}$ has to be determined either from corresponding 3D simulations or from in situ measurements, if available. Based on the observations by Pöttler [11], a parabolic decline of the remaining internal pressure $p_{i}(t)$ is assumed during the further excavation process, i.e., for t>0. Approximating the drill and blast sequence of tunnel advance, $p_{i}(t)$ is assumed as a stepwise stress release function according to Figure 2.

The numerical simulations are structured as follows: (i) application of the initial hydrostatic stress field; (ii) consideration of the initial stress release, corresponding to measured pre-displacements, i.e., the displacements due to excavation of preceding segments and of the current segment; (iii) installation of the shotcrete shell and (iv) stepwise release of the remaining internal pressure due to the subsequent excavation steps according to the stress release function. The full release of the internal pressure, i.e., $p_{i}=0$, is assumed at a progressed distance of the tunnel face of one tunnel diameter from the investigated cross section. Considering an advance length of 1 m, the internal pressure is completely released after nine excavation steps, including 8 h of rest period between two successive advance steps. Hence, the considered excavation process after installation of the shotcrete shell extends over 72 h.

### 2.3. Material Parameters for the Shotcrete Models

Material parameters for the shotcrete models are calibrated based on experimental tests conducted by Müller [21], who presented a set of experimental data on the evolution of material stiffness, uniaxial compressive strength, creep and shrinkage of shotcrete. The material parameters and the respective calibration procedure for the shotcrete models are described in detail in [8]. Some of the material parameters are common for more than one model, i.e., the Poisson’s ratio ν, the uniaxial compressive strength at the age of 1 d and 28 d, $f_{\mathrm{cu}}^{\left(1\right)}$ and $f_{\mathrm{cu}}^{\left(28\right)}$, the Young’s modulus at the age of 1 d and 28 d, $E^{\left(1\right)}$ and $E^{\left(28\right)}$, the ratios of uniaxial yield stress to uniaxial compressive strength, $f_{\mathrm{cy}}/f_{\mathrm{cu}}$, of biaxial compressive strength to uniaxial compressive strength, $f_{\mathrm{cb}}/f_{\mathrm{cu}}$, and of uniaxial tensile strength to uniaxial compressive strength, $f_{\mathrm{tu}}/f_{\mathrm{cu}}$. In lack of experimental data, the latter is assumed as $f_{\mathrm{tu}}/f_{\mathrm{cu}}=0.1$. Common parameters related to shotcrete ductility are the plastic strain at peak stress in uniaxial compression at the age of 1 h, 8 h and 24 h, $\varepsilon_{\mathrm{cpu}}^{p(1)}$, $\varepsilon_{\mathrm{cpu}}^{p(8)}$ and $\varepsilon_{\mathrm{cpu}}^{p(24)}$, and the specific mode I fracture energy at the age of 28 d, $G_{\mathrm{fI}}^{\left(28\right)}$. Common parameters related to shrinkage are the ultimate shrinkage strain, $\varepsilon_{\infty }^{\mathrm{shr}}$, the shrinkage half time, $\tau_{\mathrm{shr}}$ (SCDP model and Meschke model) and $t_{\mathrm{shr}}^{50}$ (Schädlich model), and the humidity dependent parameter $k_{h}$.

Time-dependent stiffness and creep behavior of the SCDP model, described by a modified version of the solidification theory by Bažant and Prasannan [10], are governed by the four compliance parameters $q_{1}$, $q_{2}$, $q_{3}$ and $q_{4}$, provided in Table 1. The softening behavior of the SCDP model is regularized by means of the crack-band theory [22], based on the characteristic element length, the specific mode I fracture energy $G_{\mathrm{fI}}$ and the uniaxial tensile strength $f_{\mathrm{tu}}$. The regularization scheme is described in detail in [23]. Experimental data by Brameshuber and Hilsdorf [24] indicate that the temporal evolution of the specific mode I fracture energy can be considered approximately proportional to the increase of strength. Since the regularization scheme depends only on the ratio of fracture energy to tensile strength, the softening modulus can be computed from the values determined at the age of 28 d, i.e., $G_{\mathrm{fI}}^{\left(28\right)}$ and $f_{\mathrm{tu}}^{\left(28\right)}$. In lack of experimental data by Müller on the specific mode I fracture energy, $G_{\mathrm{fI}}^{\left(28\right)}=0.1N/\mathrm{mm}$ is assumed for all models, which is in good agreement with commonly assumed values.

The creep behavior of the Meschke model is governed by the viscosity parameter η. Parameters $\Delta t_{E}$ and $t_{E}$ of the Meschke model control the early age evolution of the Young’s modulus. The respective parameters are summarized in Table 2.

In the Schädlich model, ψ denotes the dilatancy angle within the framework of a hardening and softening Mohr–Coulomb model. Softening material behavior is represented by a linearly decreasing compressive and tensile strength, specified by the ratios $f_{\mathrm{cfn}}$, $f_{\mathrm{cun}}$ and $f_{\mathrm{tun}}$, and the compressive fracture energy $G_{c}^{\left(28\right)}$ at the age of 28 d. The creep behavior of the Schädlich model is formulated on the basis of the Eurocode 2 creep model [25] with the parameters representing the creep half-time $t_{50}^{\mathrm{cr}}$ and the creep coefficient $\varphi^{\mathrm{cr}}$. They are specified in Table 3.

Material parameters of the SCDP model and the models by Schädlich and Meschke, which are not considered in the calibration procedure, are chosen as the default values, proposed in the papers on the respective shotcrete models. In particular, they comprise $\varepsilon_{\mathrm{cpu}}^{p(1)}$, $\varepsilon_{\mathrm{cpu}}^{p(8)}$ and $\varepsilon_{\mathrm{cpu}}^{p(24)}$ (SCDP model and Schädlich model), ψ, $f_{\mathrm{cfn}}$, $f_{\mathrm{cun}}$, $f_{\mathrm{tun}}$, $G_{c}^{\left(28\right)}$ (Schädlich model) and $\Delta t_{E}$ and $t_{E}$ (Meschke model).

**Figure 3:**
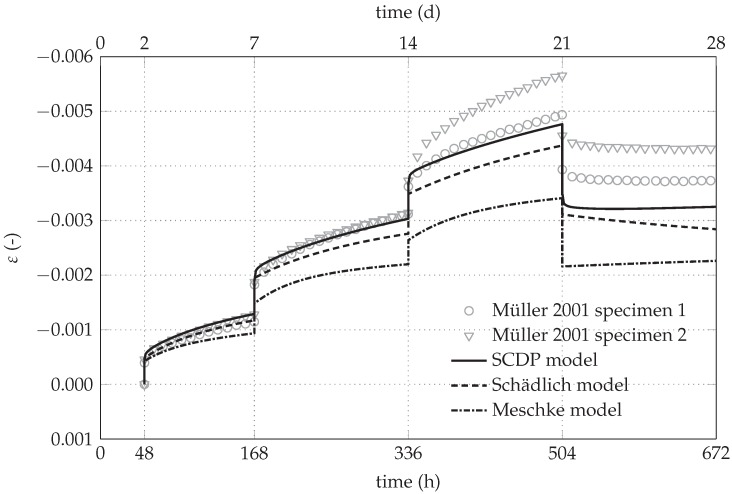
Measured and predicted evolution of the total strain of the two specimens of creep test series No. 4/2 in creep tests on unsealed specimens by Müller [21].

Figure 3 [8] depicts the evolution of the total strain of the two specimens of creep test series No. 4/2 by Müller (consistent with the notation used in [21]) and the predicted evolutions by the shotcrete models considered in the present investigation. Creep test series No. 4/2 was selected for parameter identification because of the high sustained stress levels.

For the linear-elastic shotcrete model, the hypothetical modulus of elasticity of 7000 MPa is adopted from Pöttler [11], which takes into account the time-dependent material behavior of shotcrete and time-dependent effects due to tunnel advance in a simplified manner.

### 2.4. Material Parameters for the Hoek–Brown Model for Rock Mass

The mechanical behavior of rock mass is described by the linear-elastic perfectly-plastic constitutive law with the failure criterion for rock mass by Hoek and Brown [26,27] and the Mohr–Coulomb type plastic potential function, both in the smooth version proposed by Menétrey and Willam [28]. Main simplifying assumptions inherent in the Hoek–Brown model are the neglect of hardening in the pre-peak region and softening behavior in the post peak region of the stress–strain relations.

The parameters of the Hoek–Brown model were determined from triaxial compression tests on small-scale intact rock specimens of Innsbruck quartz phyllite from drill cores sampled from the tunnel site [18]. Since the Hoek–Brown model is restricted to isotropic material behavior, average values of the identified parameters for the different loading angles in the triaxial compression tests are applied in the numerical simulations. The parameters for the Hoek–Brown model are summarized in Table 4. *E*, ν, $f_{\mathrm{cu}}$, $m_{0}$, ψ and *e* denote the Young’s Modulus, the Poisson’s ratio, the uniaxial compressive strength, the friction parameter, the dilatancy angle and the eccentricity parameter for the Menétrey–Willam formulation of the yield criterion and the plastic potential function, respectively. The down-scaling factors for the transition from intact rock to rock mass, i.e., the geological strength index GSI and the disturbance factor *D* [27], are chosen according to the geological survey at the tunnel site [18].

## 3. Finite Element Simulations of Tunnel Advance

**Figure 4:**
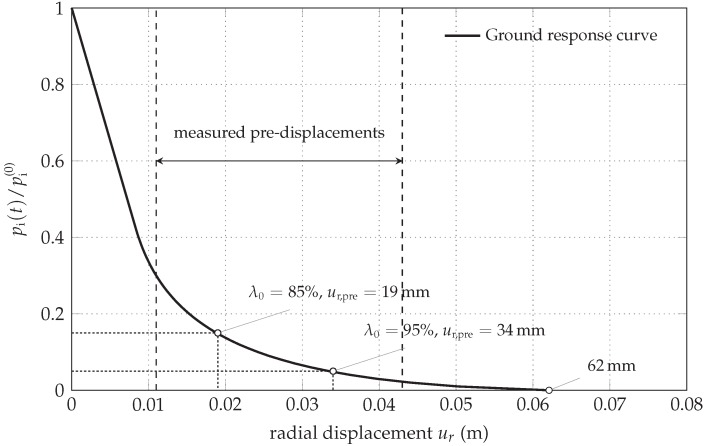
Ground response curve computed on the basis of the Hoek–Brown model.

In the 2D finite element simulations, the structural behavior of the shotcrete shell is simulated for initial stress release ratios $\lambda_{0}$ (Figure 2) of 85% and 95% at installation of the shotcrete shell. The corresponding pre-displacements at the rock-shotcrete interface are obtained from the ground response curve, computed on the basis of the Hoek–Brown model for the rock mass with the parameters according to Table 4, resulting in 19 mm and 34 mm, respectively, illustrated in Figure 4. For comparison, at the addressed tunnel site, preplaced measurement devices located approximately 1 m above the tunnel roof recorded in situ pre-displacements between 11 mm and 43 mm [18].

In addition, from the ground response curve, the maximum displacement of the tunnel surface of 62 mm at vanishing internal pressure, corresponding to the solution without the supporting shotcrete shell, is obtained. It can serve in the subsequent investigation as a reference for assessing the supporting effect achieved by the shotcrete shell.

The simulations are conducted by the finite element software Abaqus/Standard v6.14-1 (Dassault Systèmes: Providence, RI, USA) [29]. The constitutive models for rock and shotcrete are implemented as user material (UMAT) subroutines, providing the updated stress and consistent tangent stiffness at each integration point within the framework of the return mapping algorithm.

### 3.1. Predicted Displacements of the Shotcrete Shell

The computed uniform radial displacement along the tunnel perimeter at the shotcrete-rock interface is used for comparing the influence of the shotcrete models on the structural behavior of the shotcrete shell. To this end, a period of 408 h (17 d) after installation of the shotcrete shell is considered. It includes the duration of the nine excavation and securing steps, extending over 72 h (3 d) according to Figure 2, plus additional 336 h (14 d) for the completed tunnel. The predicted evolution of the radial displacement includes (i) the pre-displacement, which is related to the initial stress release before installation of the shotcrete shell; (ii) the instantaneous and time-dependent displacement during the subsequent nine excavation and securing steps and (iii) the time-dependent displacement of the completed tunnel, resulting from creep and shrinkage of the shotcrete.

**Figure 5:**
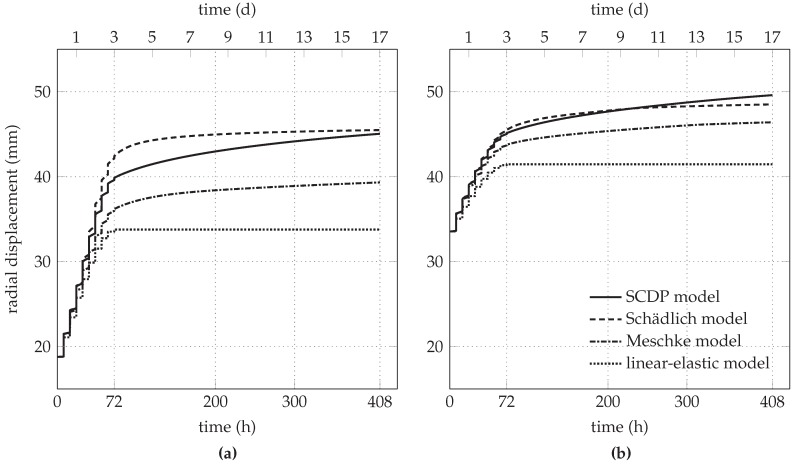
Comparison of the predicted radial displacement at the shotcrete-rock interface for initial stress release ratios of (**a**) 85% and (**b**) 95%.

Figure 5a shows a comparison of the predicted evolution of the radial displacement at the shotcrete-rock interface for the initial stress release ratio of 85%. It can be seen that the linear-elastic shotcrete model predicts the smallest displacement, i.e., the stiffest response of the shotcrete shell. In contrast, the Schädlich model predicts the largest instantaneous displacement during the nine excavation and securing steps, which is due to the larger plastic strains predicted by this shotcrete model. It will be shown in Section 3.2 that this is a consequence of the employed Mohr–Coulomb yield criterion. Compared to the Schädlich model, the SCDP model predicts smaller instantaneous displacements during the excavation and securing steps, but a stronger increase of the displacement due to creep, approaching the radial displacement of the Schädlich model by the end of the investigated time period at t=17 d. Displacements predicted by the Meschke model are in between the results using the SCDP model and the Schädlich model on the one hand, and the linear-elastic model on the other hand.

The respective comparison of the predicted evolution of the radial displacement for the initial stress release ratio of 95% is shown in Figure 5b. Due to the higher initial stress release, smaller instantaneous displacements during the excavation and securing steps are predicted by all models, compared to the results shown in Figure 5a. Again, the SCDP model predicts the strongest increase of the time-dependent displacement due to creep, which is in good agreement with the simulations of the creep tests [21] by the investigated shotcrete models, shown in Figure 3.

**Figure 6:**
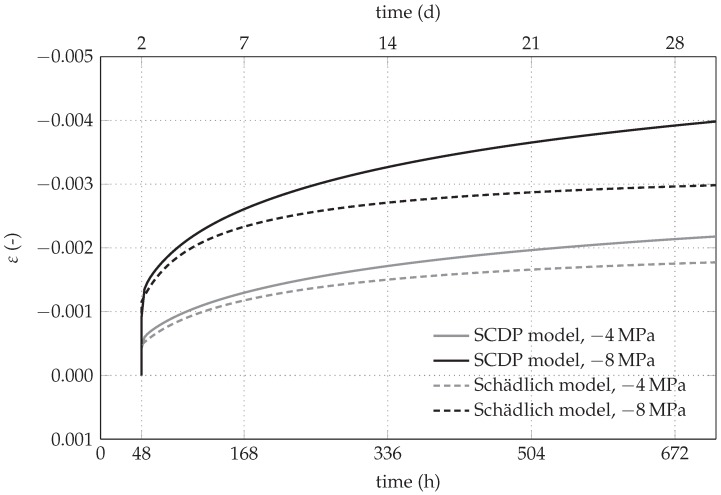
Comparison of the creep behavior, based on the evolution of the total strain, predicted by the SCDP model and the Schädlich model, for sustained compressive stresses of -4 MPa and -8 MPa.

The differences between the SCDP model and the Schädlich model concerning the predicted time-dependent displacements need some further explanations. The creep law of the Schädlich model is formulated by means of a temporally decreasing ultimate creep strain, which is related to the evolution of the Young’s modulus. As a consequence, creep slows down already several days after casting of the shotcrete shell. Since the SCDP model [8] is based on a modified version of the solidification theory by Bažant and Prasannan [10], which is partially based on the log-double power law by Bažant and Chern [30], the evolution of the long-term creep strain is unbounded. The different creep behavior, predicted by the Schädlich model and the SCDP model, is illustrated on the basis of uniaxial creep tests on shotcrete specimens with two different levels of sustained compressive stresses of -4 MPa and -8 MPa in Figure 6. It can be seen that the creep strains predicted by the SCDP model increase faster than the ones predicted by the Schädlich model and the differences between the predicted creep strains increase with increasing stress levels.

At the tunnel site, radial displacements of the shotcrete shell were recorded by geodetic measurements in the range of 14 mm to 38 mm two weeks after excavation of the corresponding segment. According to Figure 5a, in simulations based on the initial stress release ratio of 85%, displacements induced by the subsequent excavation process after installation of the shotcrete shell are predicted in the range of 15 mm (linear-elastic model) to 27 mm (Schädlich model and SCDP model). For the initial stress release ratio of 95%, the predicted displacements induced by the subsequent excavation process after installation of the shotcrete shell of 8 mm (linear-elastic model) to 16 mm (SCDP model), according to Figure 5b, are smaller than the measured displacements. Thus, the initial stress release ratio of 85% results in reasonable agreement of the predicted displacements after installation of the shotcrete shell with the geodetic measurements.

### 3.2. Predicted Stress Response

The degree of loading of the shotcrete shell is evaluated by means of the stress state at the integration point located closest to the free surface of the shotcrete shell. For each shotcrete model, the evolution of the principal stress components of the biaxial stress state, i.e., the circumferential stress $\sigma_{C}$ and the longitudinal stress $\sigma_{L}$ perpendicular to the considered cross-section, are investigated.

**Figure 7:**
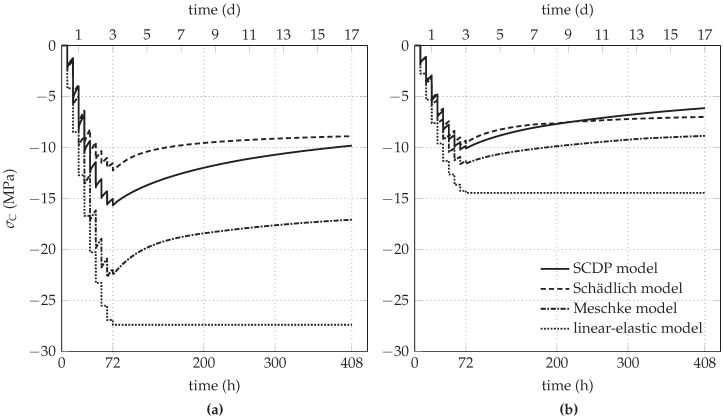
Predicted evolution of the circumferential stress in the shotcrete shell for initial stress release ratios of (**a**) 85% and (**b**) 95%.

Figure 7a shows the predicted evolution of $\sigma_{C}$ for the initial stress release ratio of 85%. Both the linear-elastic shotcrete model and the Meschke model predict circumferential stresses that are larger than the experimentally determined strength of young shotcrete. In case of the Meschke model, this shortcoming is attributed to the viscoplastic formulation, which is characterized by limiting rate-dependent material behavior, in particular stress relaxation, to stress states outside the yield surface. In contrast, the SCDP model and the Schädlich model predict considerably smaller circumferential stresses, resulting from fast stress relaxation of the young shotcrete.

**Figure 8:**
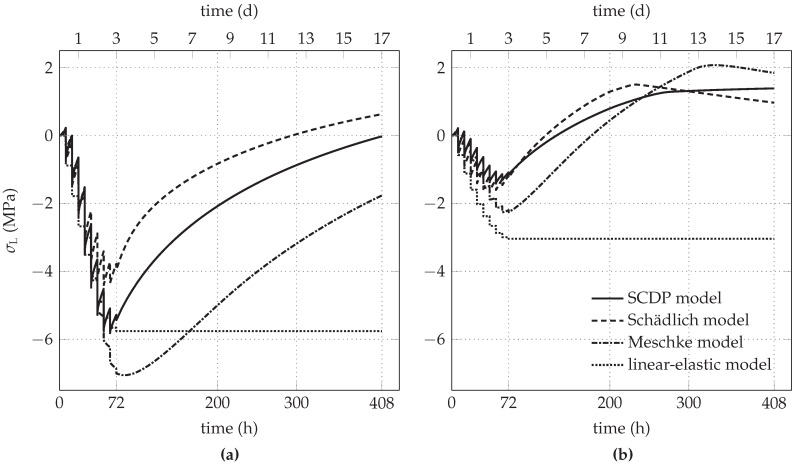
Predicted evolution of the longitudinal stress in the shotcrete shell for initial stress release ratios of (**a**) 85% and (**b**) 95%.

**Figure 9:**
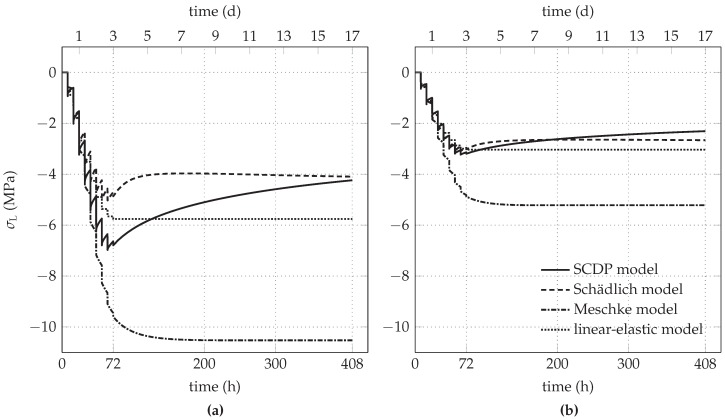
Predicted evolution of the longitudinal stress, if shrinkage of shotcrete is neglected, for initial stress release ratios of (**a**) 85% and (**b**) 95%.

The predicted evolution of $\sigma_{L}$ for the initial stress release ratio of 85% is shown in Figure 8a. Because of the plane strain conditions, the strain in longitudinal direction of the tunnel, induced by shrinkage of shotcrete, is prevented by an evolving tensile stress. Hence, the nonlinear shotcrete models predict a decreasing compressive longitudinal stress due to shrinkage, gradually changing to a tensile stress in the case of the Schädlich model. The effect of shrinkage on the evolution of the longitudinal stress is also reported in [16]. For comparison, Figure 9a shows the evolution of $\sigma_{L}$, if shrinkage is neglected in the three models. Expectedly, in this case the longitudinal stresses remain in the compressive regime.

For the initial stress release ratio of 95%, the evolution of $\sigma_{C}$ and $\sigma_{L}$ is shown in Figure 7b and Figure 8b, respectively. The higher initial stress release results in smaller circumferential stresses, accompanied by tensile stresses in longitudinal direction due to shrinkage. For all three nonlinear shotcrete models, the tensile stresses in longitudinal direction attain the tensile strength, leading to tension softening. The SCDP model and the Meschke model predict a minor effect of softening, indicated for the SCDP model by the maximum value of the isotropic scalar damage variable of ω= 2% only and for the Meschke model by a reduction of the uniaxial tensile yield stress of 13% with respect to the tensile strength. In contrast, in the case of the Schädlich model, the uniaxial tensile strength is reduced due to softening by 27%. Figure 9b depicts the evolution of the longitudinal stress, if shrinkage is neglected in the shotcrete models.

**Figure 10:**
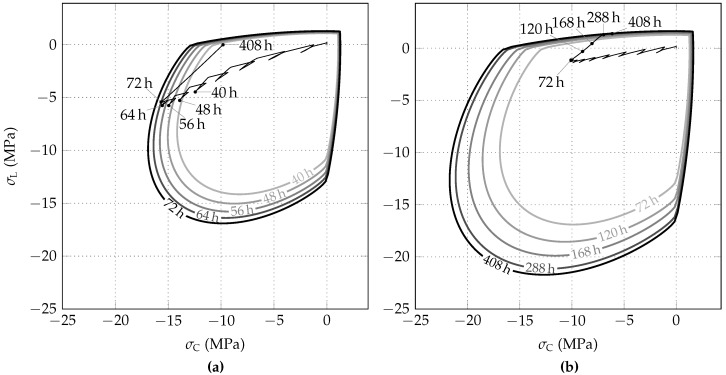
Evolution of biaxial strength envelopes of shotcrete and stress paths, predicted by the SCDP model for initial stress release ratios of (**a**) 85% and (**b**) 95%.

The utilization of the shotcrete shell is visualized by plotting the evolution of the biaxial strength envelope together with the stress path. Figure 10a,b show the evolution of the biaxial strength envelope and the stress paths, predicted by the SCDP model for the initial stress release ratios of 85% and 95%, respectively. In the case of 85% initial stress release, during the last steps of the excavation process (t≤72 h), the stress state approaches the biaxial strength envelope, whereas, for t>72 h, the compressive stresses are reduced due to stress relaxation and shrinkage. Thus, the outermost biaxial strength envelope approached by the stress state is the one at t=72 h. In contrast, considering the initial stress release ratio of 95%, the stress path approaches the strength envelope due to shrinkage in the tensile-compressive region towards the end of the time period considered. Thus, the outermost biaxial strength envelope, relevant for the numerical simulation, is the one at t=408 h.

**Figure 11:**
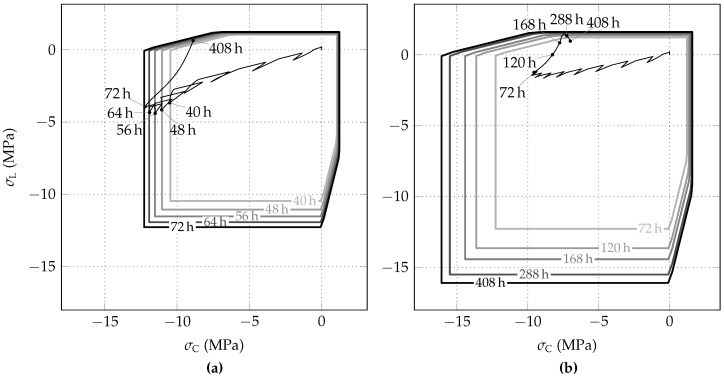
Evolution of biaxial strength envelopes of shotcrete and stress paths, predicted by the Schädlich model for initial stress release ratios of (**a**) 85% and (**b**) 95%.

The evolution of the biaxial strength envelope of shotcrete and the stress path, predicted on the basis of the Schädlich model, are shown in Figure 11a,b for the initial stress release ratios of 85% and 95%, respectively. Contrary to the SCDP model, for the initial stress release ratio of 85%, the stress state attains the biaxial strength envelope. Hence, compressive softening behavior occurs as a consequence of the employed Mohr–Coulomb yield criterion, which underestimates the biaxial compressive strength by assuming it equal to the uniaxial compressive strength. However, it follows from Figure 11a, that the predicted compressive softening is negligible.

**Figure 12:**
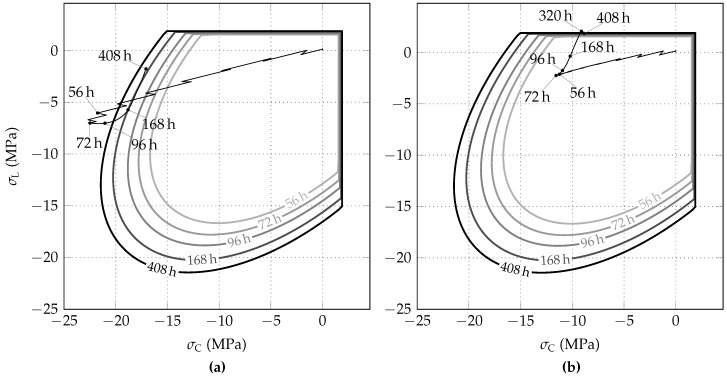
Evolution of biaxial strength envelopes of shotcrete and stress paths, predicted by the Meschke model for initial stress release ratios of (**a**) 85% and (**b**) 95%.

Figure 12a,b show the evolution of the biaxial strength envelope and stress path, predicted by the Meschke model for the initial stress release ratios of 85% and 95%, respectively. Note that temporally limited stress states located outside of the biaxial strength envelope are typical for the theory of viscoplasticity, applied in the Meschke model.

### 3.3. Long-Term Behavior Employing the SCDP Model

Since in contrast to the shotcrete models by Meschke and by Schädlich, the SCDP model predicts an unbounded evolution of the long-term creep strain, special attention is paid to the long-term behavior predicted by the SCDP model. For this purpose, the investigated time period is extended to two years, i.e., 17,520 h.

**Figure 13:**
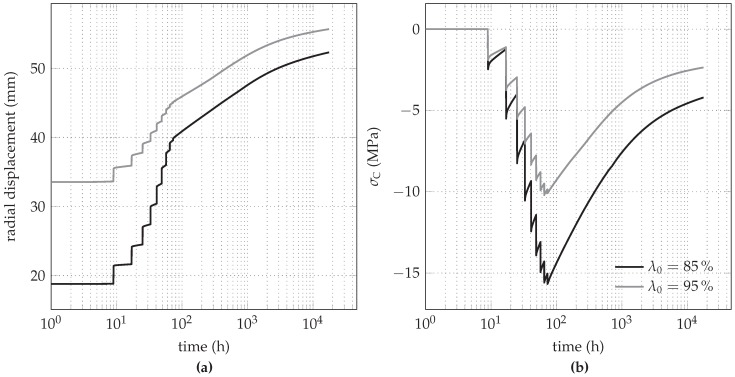
Long-term behavior predicted by the SCDP model for both initial stress release ratios: evolution of (**a**) the radial displacement and (**b**) the circumferential stress.

In Figure 13, the long-term evolutions of the radial displacement and the circumferential stress are shown in a semi-logarithmic scale for both initial stress release ratios. After two years, radial displacements of 52 mm and 55 mm are obtained for the initial stress release ratios of 85% and 95%, respectively. Hence, despite the different initial stress release ratios, resulting in different pre-displacements, the predicted long-term displacements are getting closer. In contrast to the radial displacements, after two years, the circumferential stresses of -4.2 MPa ($\lambda_{0}=$ 85%) and -2.35 MPa ($\lambda_{0}=$ 95%) are still quite different, but are approaching each other slowly.

## 4. Conclusions

Based on finite element simulations of deep tunnel advance, the influence of the constitutive model for shotcrete on the predicted structural behavior of the shotcrete shell was evaluated. For this purpose, different shotcrete models were employed in a benchmark study derived from a stretch of the Brenner Basetunnel, driven in Innsbruck quartz phyllite by the New Austrian Tunneling Method. In particular, the linear-elastic shotcrete model with a reduced Young’s modulus proposed by Pöttler, the viscoplastic shotcrete model by Meschke, the viscoelastic-plastic shotcrete model with the creep model according to the Eurocode 2 by Schädlich and Schweiger and the shotcrete damage plasticity (SCDP) model, characterized by representing aging and creep by the solidification theory by Bažant and Prasannan and by the shrinkage model by Bažant and Panula, were considered.

Since the focus of this contribution was the evaluation of different shotcrete models, the following assumptions for the numerical model were made: The 3D problem of tunnel advance was approximated by a 2D plane strain model, employing the commonly used Hoek–Brown model for describing the material behavior of the surrounding rock mass. In addition, the assumed hydrostatic initial stress state and the considered full-face excavation allowed the reduction to an axisymmetric problem with respect to the tunnel axis. In the 2D finite element simulations, two different initial stress release ratios of 85% and 95% at installation of the shotcrete shell were considered according to in situ measurements for the addressed tunnel stretch. The influence of the shotcrete models on the predicted structural behavior of the shotcrete shell was compared with respect to the evolution of the predicted radial displacement and the circumferential and longitudinal stresses in the shell. From the results, the following conclusions are drawn:

- The assumption of linear-elastic material behavior of shotcrete with a reduced Young’s modulus to account for time-dependent effects in a simplified manner results in an overestimation of the stiffness of the shotcrete shell compared to shotcrete models, which consider time-dependent material behavior. Certainly, the results could be improved by assuming a smaller value of the reduced Young’s modulus; however, estimation of the latter relies on engineering judgement.
- Compared to the SCDP model and the Schädlich model, a very stiff response of the shotcrete shell is also predicted by the Meschke model, which is caused by describing time-dependent shotcrete behavior by a viscoplastic formulation. A shortcoming of the latter is the restriction of creep to stress states located outside of the elastic domain.
- The SCDP model and the Schädlich model predict similar results concerning the short-term response of the shotcrete shell. Concerning the predicted long-term response of the shotcrete shell, substantially larger creep strains are predicted by the SCDP model because of the unbounded long-term creep strain of the employed modified version of the solidification theory by Bažant and Prasannan, which is partially based on the log-double power law by Bažant and Chern.
- Shrinkage has a considerable influence on the overall behavior of the shotcrete shell, as it counteracts the compressive longitudinal stresses, induced by the excavation process, due to evolving tensile stresses resulting from constrained deformations in longitudinal direction of the tunnel. Because of shrinkage, a higher initial stress release ratio does not necessarily lead to a lower degree of loading of the shotcrete shell. As demonstrated for the high initial stress release ratio of 95%, the assumed zero-strain condition in the longitudinal direction results in tensile stresses up to the tensile strength and, consequently, in tension softening.

## Figures and Tables

**Table 1:** Material parameters for the SCDP model.

$q_{1}$ $\left({\mathrm{MPa}}^{-1}\right)$	$q_{2}$ $\left({\mathrm{MPa}}^{-1}\right)$	$q_{3}$ $\left({\mathrm{MPa}}^{-1}\right)$	$q_{4}$ $\left({\mathrm{MPa}}^{-1}\right)$	ν (-)	$E^{\left(1\right)}$ (MPa)	$f_{\mathrm{cu}}^{\left(1\right)}$ (MPa)	$f_{\mathrm{cu}}^{\left(28\right)}$ (MPa)	$f_{\mathrm{cy}}/f_{\mathrm{cu}}(-)$
59.5 × 10^-6^	269.82 × 10^-6^	3.84 × 10^-6^	54.2 × 10^-6^	0.21	7690	8.72	16.8	0.1
$f_{\mathrm{cb}}/f_{\mathrm{cu}}(-)$	$f_{\mathrm{tu}}/f_{\mathrm{cu}}(-)$	$\varepsilon_{\infty }^{\mathrm{shr}}$ (-)	$k_{h}$ (-)	$\tau_{\mathrm{shr}}$ (-)	$\varepsilon_{\mathrm{cpu}}^{p(1)}$ (d)	$\varepsilon_{\mathrm{cpu}}^{p(28)}$ (-)	$\varepsilon_{\mathrm{cpu}}^{p(24)}$ (-)	$G_{\mathrm{fI}}^{\left(28\right)}(N/\mathrm{mm})$
1.16	0.1	-0.0019	1.0	32	-0.03	-0.0015	-0.0007	0.1

**Table 2:** Material parameters for the Meschke model.

$E^{\left(1\right)}(\mathrm{MPa})$	$E^{\left(28\right)}(\mathrm{MPa})$	ν (-)	$f_{\mathrm{cu}}^{\left(1\right)}(\mathrm{MPa})$	$f_{\mathrm{cu}}^{\left(28\right)}(\mathrm{MPa})$	$f_{\mathrm{cy}}/f_{\mathrm{cu}}(-)$	$f_{\mathrm{cb}}/f_{\mathrm{cu}}(-)$	$f_{\mathrm{tu}}/f_{\mathrm{cu}}(-)$
7690	11580	0.21	8.72	16.8	0.1	1.16	0.1
η (h)	$\varepsilon_{\infty }^{\mathrm{shr}}$ (-)	$k_{h}$ (-)	$\tau_{\mathrm{shr}}$ (d)	$\Delta t_{E}$ (h)	$t_{E}$ (h)	$G_{\mathrm{fI}}^{\left(28\right)}$ (N/mm)	
16.1	-0.0019	1.0	32	6	8	0.1	

**Table 3:** Material parameters for the Schädlich model.

$E^{\left(1\right)}$ (MPa)	$E^{\left(28\right)}$ (MPa)	ν (-)	ψ ${(}^{{}^{\circ}})$	$t_{50}^{\mathrm{cr}}$ (h)	$\varphi^{\mathrm{cr}}$ (-)	$f_{\mathrm{cu}}^{\left(1\right)}$ (MPa)	$f_{\mathrm{cu}}^{\left(28\right)}$ (MPa)	$f_{\mathrm{cu}}^{\left(28\right)}$ (MPa)	$f_{\mathrm{cy}}/f_{\mathrm{cu}}(-)$
7690	11580	0.21	0	24	1.21	8.72	16.8	1.68	0.1
$f_{\mathrm{cfn}}$ (-)	$f_{\mathrm{cun}}$ (-)	$f_{\mathrm{tun}}$ (-)	$\varepsilon_{\infty }^{\mathrm{shr}}$ (-)	$t_{50}^{\mathrm{shr}}$ (d)	$\varepsilon_{\mathrm{cpu}}^{p(1)}$ (-)	$\varepsilon_{\mathrm{cpu}}^{p(8)}$ (-)	$\varepsilon_{\mathrm{cpu}}^{p(24)}$ (-)	$G_{\mathrm{fI}}^{\left(28\right)}\left(N/\mathrm{mm}\right)$	$G_{c}^{\left(28\right)}$ (N/mm)
0.1	0.1	0.1	-0.0015	8.3	-0.03	-0.0015	-0.0007	0.1	30

**Table 4:** Material parameters of Innsbruck quartz phyllite for the Hoek–Brown model.

E(MPa)	ν (-)	$f_{\mathrm{cu}}\;(\mathrm{MPa})$	$m_{0}$ (-)	ψ ${(}^{{}^{\circ}})$	e (-)	GSI (-)	D (-)
56,670	0.21	42	12	11.6	0.51	40	0
